# Rehabilitation needs in individuals with knee OA in rural Western Cape, South Africa: an exploratory qualitative study

**DOI:** 10.1017/S1463423620000043

**Published:** 2020-03-19

**Authors:** Marisa Coetzee, Marlie Giljam-Enright, Linzette Deidre Morris

**Affiliations:** 1 Senior Research Assistant and PhD Student, Division of Physiotherapy, Department of Health and Rehabilitation Sciences, Faculty of Medicine and Health Sciences, University of Stellenbosch, Tygerberg 7505, Republic of South Africa; 2 Lecturer and Clinical Educator, Division of Physiotherapy, Department of Health and Rehabilitation Sciences, Faculty of Medicine and Health Sciences, University of Stellenbosch, Tygerberg 7505, Republic of South Africa; 3 Assistant Professor, Department of Physical Therapy & Rehabilitation Science, College of Health Sciences, QU Health, Qatar University, Doha, Qatar

**Keywords:** knee, needs, osteoarthritis, PHC, rehabilitation

## Abstract

**Background:**

Current clinical practice guidelines have suggested that each individual with knee osteoarthritis (OA) should receive three core treatments from their health care provider. These core treatments comprise of exercise, education and weight loss. Identification of the health care and rehabilitation needs of individuals with knee OA in rural areas are imperative for focusing service delivery in a specific context in order to empower the individual. The aim of this study was to explore the rehabilitation needs of individuals with knee OA living in rural Western Cape in order to identify the gaps in services offered and inform rehabilitation programmes in these settings.

**Methods:**

Semi-structured in-depth individual interviews were performed on 16 individuals with knee OA living in rural settings of the Western Cape, South Africa. A deductive data analysis approach was used and the needs of the individuals were identified and categorised for interpretation and comparison with the reported services received.

**Findings:**

The three major themes identified were ‘I would like to know more’, ‘There’s not much support from the clinic’ and ‘I don’t feel myself anymore’. These themes relate to the lack of disease-specific education, barriers in the health systems and service delivery resulting in individuals lack of self-worth and poor mental wellbeing. The results revealed the integral relationship between health care systems, service delivery and the effect on patient wellbeing.

**Conclusion:**

The rehabilitation needs of individuals with knee OA in rural areas advocates for addressing barriers in rural primary health care system such as adequate human resources, referral systems and continuity of care. This will allow for a comprehensive, person-centred and context-specific multidisciplinary approach focused on empowering individuals with knee OA through disease-specific education, improving functional participation and symptom management strategies. This could improve the social inclusion and mental wellbeing of individuals living with knee OA.

## Introduction

Osteoarthritis (OA) is one of the leading causes of chronic joint-related functional disability for individuals in developed, as well as developing countries (Spencer, Degu, Kalkidan, Solomon, Christiana, Nooshin, Hedayat *et al.*, 2018). OA typically affects individuals over the age of 45 years who are still productive members of their communities and the economy. In addition, the functional decrease resulting from OA could lead to loss of employment and overall decreased quality of life (Prieto-Alhambra *et al.*, 2014).

Current clinical practice guidelines suggest that the health care provider should include three core treatments in the management plan of the patient. These core treatments should consist of education, exercises and weight loss therapy (if required) (Conaghan *et al.*, 2008; Hochberg *et al.*, 2012; Nelson *et al.*, 2014; Glyn-Jones *et al.*, 2015). Ideally, the core services would be delivered by a multidisciplinary team (MDT) which consists of the doctor, nurse, physiotherapist, occupational therapist and dietician, all focusing on their respective areas of expertise. However, the primary health care (PHC) system in South Africa, especially in the rural areas, is not readily equipped with full time MDTs, and patients are often only seen by one or two of the members in the team (Bateman, 2012; Lalkhen and Mash, 2015). In addition, underutilisation of rehabilitation services and the lack of adherence to evidence-based guidelines is a concern previously found in PHC settings of South Africa for patients suffering from musculoskeletal disorders (Major-Helsloot *et al.*, 2014).

Due to the recent increase in non-communicable diseases amongst South Africans, along with the ageing population influenced by multi-morbidity, it has become imperative to empower the individual in their health care management by addressing their needs and ensuring a person-centred approach (Mayosi *et al.*, 2009; Gómez-Olivé *et al.*, 2013; Lalkhen and Mash, 2015). This can be achieved by delivering self-management programmes that is specifically tailored for the individual within their setting. Therefore, the aim of this study was to explore the rehabilitation needs of individuals with knee OA living in rural Western Cape in order to identify the gaps in services offered and inform rehabilitation programmes in these settings.

## Method

This section is reported according to the Standards for Reporting Qualitative Research as proposed by O’Brien *et al.* (2014) and the Consolidated Criteria for Reporting Qualitative Research as proposed by Tong *et al.* (2007).

### Study design

A descriptive and exploratory qualitative research method with a phenomenological approach was used for this study. This approach was chosen to obtain insight into the current management strategies and services received by the health care personnel for knee OA and to gain insight into the needs and experience of these individuals with regard to the management and rehabilitation of their knee OA and determine what is at the core of the rural rehabilitation for individuals with knee OA (Creswell *et al.*, 2007).

### Study setting

The study was performed in three randomly selected rural areas of the Western Cape, South Africa. Rural areas in South Africa have been defined as areas of sparsely populated towns where individuals are dependent on natural resources and agricultural activities for survival (Republic of South Africa, Department of Treasury, 2012). Typically, these towns are situated far from the metropolitan and have limited services available. In the Western Cape, these segregated areas originated through missionary work and resulted in generations of families who live in these areas and formed close-knit communities with strong cultural beliefs (Western Cape Government, 2014). The individuals within these areas are often dependent on the satellite clinics that are operating only twice to three times a week with visiting rehabilitation personnel, physicians and community health care workers.

### Sample

This study used a non-probability sampling method due to the lack of a central or community-based patient registry for patients with OA. Health care-seeking individuals and potential participants were approached in the waiting area of the clinics, after which a snowball sampling technique was used to obtain the names of potential participants with knee pain or knee OA. The clinic visit for recruitment was scheduled early morning as the highest number of health care users was present at that time. These individuals were approached and permission to read their clinical files was obtained. Individuals who met the inclusion criteria were then invited to take part in the study. *Inclusion criteria*: individuals diagnosed with knee OA by either the physician, registered nurse or the physiotherapist (as per their file at the clinic) and who were aged 45 years and older were invited to take part in the study. *Exclusion criteria*: potential participants were excluded if they were diagnosed with other conditions that could influence their functional abilities (such as stroke, gout or rheumatoid arthritis) or unclear diagnosis in their file.

### Researcher characteristics

The research and interviews were conducted solely by the lead author, a qualified female physiotherapist with eight years of general practice experience and two years’ experience working in rural communities. The researcher had some experience in conducting qualitative interviews and was informally trained by a peer qualitative researcher prior to conducting the study. The researcher had no previous contact or relationship with the participants in these areas. The participants were aware of the occupation of the researcher as well as the fact that the researcher was conducting the research for the purpose of completing a master’s degree. The researcher is fluent in the home language of the participants and conducted the interviews in this language. Having worked in rural settings before, the researcher had assumptions regarding service delivery and patient participation based on experience. However, the researcher attempted to stay open-minded and explorative for the entire data collection and analysis process. The researcher is a White Afrikaans-speaking female born in South Africa, living in the Western Cape and a qualified physiotherapist.

### Data collection instruments and methods

Informed consent and permission to audio record the interviews were obtained from the participants. In-depth, face-to-face semi-structured individual interviews were used as a method for data collection during March and April 2018. The interview schedule was developed using the International Classification of Functioning, Disability and Health (ICF) as well as the Osteoarthritis Quality Indicator Questionnaire as a framework to obtain information about the current services patients receive at their PHC facility and the needs of these individuals (World Health Organization, 2003; Osterås *et al.*, 2013). A pilot interview was used to test the interview schedule which was not used for analysis or included in the results. The interview schedule was used as a guideline for conversation, and field notes as well as a diary were used by the researcher to document aspects of the interviews and the communities that were not spoken on record. Open-ended questions such as ‘How has the knee OA affected your life’ and ‘What are you currently receiving as treatment for your knee OA’ were used to start the interview followed by prompting of specific components such as their knowledge on OA, the role of exercise and diet as well as what they felt they would like to receive. Demographic information (age, sex, level of education, occupation and years diagnosed with knee OA) was collected using a separate questionnaire. Interviews were conducted at a place which was chosen by the participant (mostly at their homes but some at the clinic or the local library) and the duration of the interview ranged between 25 and 50 min. The researcher was accompanied by a community health care worker when approaching individuals and when doing a home visit to conduct the interview in order to assist with community introduction. Only the researcher and the participant were present during the interview process. Due to time constraints member checking was done during the interviews, as described by Shenton (2004) and Britten (2006), in order to confirm that the interpretation by the researcher reflects the true meaning of the data collected from the participant. Interviews were performed until data saturation was reached and no new concepts were introduced during the interview process.

### Data processing and analysis

The recorded interviews were transcribed verbatim, validated by the researcher and translated to English (validation after translation took place during the coding process to ensure that the true meaning is reflected) after which the content was coded on qualitative data software using a deductive coding approach and a framework analysis. A deductive coding approach with a framework analysis (Hsieh and Shannon, 2005; Pope *et al.*, 2006) was used for the analysis process. The framework analysis involves the creation of a structure for analysis, based on the objectives of the study and the interview schedule, by which coding and sorting of information will take place (Rabiee, 2004; Pope *et al.*, 2006). Even though this is an organised approach, it is still flexible to be guided by the data (Pope *et al.*, 2006). Using the ICF framework to inform the interview schedule, the use of a deductive analysis was chosen to ensure that all the possible contextual factors (environmental and personal) were analysed according to the topics covered during the interview. The primary investigator then created a codebook based on the interview structure and the objectives of the study, and added extra codes as more themes emerged during the coding process. The coding of the first three interviews was done by the researcher and one of the co-authors (MGE), to allow for validation of the coding and to reach consensus on the interpretation of the content and common emerging themes.

### Ethics

Permission to conduct this study was provided by the Stellenbosch University Health Research Ethics Committee in November 2017 (Ref no S17/09/172) as well as the Department of Health Western Cape Government (Ref WC_201712_003). The authors assert that all procedures contributing to this work comply with the ethical standards of the relevant national and institutional guidelines and with the Helsinki Declaration of 1975, as revised in 2008.

## Results

A total of 18 individuals (6 individuals per rural setting) were interviewed for this study. The first two interviews were excluded from data analysis as they were used as pilot interviews and leading questions were changed upon reflection after the interviews. Exclusion of other potential participants (*n* **=** 19) was for the following reasons: below the age of 45 years (*n* **=** 3), had rheumatoid arthritis (*n* **=** 5), had gout (*n* **=** 3), had a stroke (*n* **=** 1), had low back pain with referral to the knee (*n* **=** 1) or no clear diagnosis in the file/the file could not be found (*n* **=** 6). All of the included participants were native Afrikaans-speaking individuals. Demographic data of the participants are presented in Table 1.

**Table 1.** Demographic and disease-related information of study participants

		Individual (*n*=16)
Age of participants in years	Median (range)	67.5 (49 – 84)
Gender of participants	Female (%)	15 (93.75)
	Male (%)	1 (6.25)
Level of education (grade completed)	Median (range)	9 (0–12)
	Able to read (%)	15 (93.75)
Currently working	Yes (%)	1 (6.25)
	No (%)	15 (93.75)
Current/previous type of employment	Nursing assistant (%)	1 (6.25)
	None (%)	2 (12.5)
	Care taker (%)	1 (6.25)
	Catering (%)	4 (25)
	Domestic (%)	4 (25)
	Factory (%)	4 (25)
	Farm work (%)	3 (18.75)
	Sewing (machine) (%)	1 (6.25)
Years living with OA	1–5 (%)	3 (18.75)
	6–10 (%)	2 (12.5)
	11–15 (%)	2 (12.5)
	16–20 (%)	0 (0)
	>20 (%)	9 (56.25)
Currently receiving rehabilitation	Yes (%)	2 (12.5)
	No (%)	14 (87.5)
Have consulted (*) for their knee OA	*Doctor (%)	14 (87.5)
	*Physiotherapist (%)	9 (56.25)
	*Occupational therapist (%)	0 (0)
	*Nurse (%)	16 (100)
	*Dietician (%)	2 (12.5)
	*Specialist (%)	5 (31.25)

As presented in Table 1, all of the participants (*n* **=** 16) consulted the nurse at the clinic for their knee OA and the majority had a consultation with the doctor (*n* **=** 14). During these consultations, all of the participants received at least one form of pharmacological intervention and half of them (*n* **=** 8) received some education and exercise guidance.

The main themes identified during the interviews were the participants’ need to know more disease-specific information (‘I would like to know more’), the lack of support from the health care system (‘There’s not much support from the clinic’) as well as a need to feel themselves again (‘I don’t feel myself anymore’).

Quotes from the participants are followed by the years diagnosed to give more insight into the time they have spent struggling with the disease with limited knowledge and misconceptions, not having the appropriate support.

### ‘I would like to know more’

The participants expressed a pressing need for knowledge on the condition and the expected disease progression. Only half of the participants recalled receiving education relating to OA of which two participants received information on the condition itself. This lead to the perception that OA is due to old age and that little can be done for them. They were uncertain of the effect that their daily activities would have on their symptom progression and felt they would like to have more guidance on what they should do or things to avoid. In general, participants were oblivious to what OA is or how it could be treated and thought that they were destined to live a life with disability.

‘Okay, the doctor tells me they can’t do anything about it. It is just a lifelong pain that you have to bear … I would like to know more. I’ve had it for so long now, more than 20 years … They told me the one was going to infect the other one later on. Is it true?’ **(>20 years diagnosed)**

‘I only want to receive good treatment …. And to tell me what the cause is. You see? I want to know about things like that.’ **(>20 years diagnosed)**

Most participants did not know which treatment options are available to them. Advice on adhering to healthy diets was given, but lacked detail such as the exact elements of a healthy diet, especially in their rural context. When asked about the current treatment they received, participants were focused on listing the medication prescribed from the doctor, with minimal insight into other forms of pain management or rehabilitation. One participant was merely told that the next option for her is to have surgery.

‘But just give me a prescription of how I should have to lose weight, I’ll do it too, but just prescribe on how I should eat, I will do it.’ **(>20 years diagnosed)**

‘But no, they just said come to the clinic to get your appointment when to go to hospital. Now I have to go to hospital and I don’t know what for. It doesn’t sound right so now I don’t want to go.’ **(5 years diagnosed)**

In general, the majority of participants felt ill-equipped to manage their condition with anything other than pain medication and was unaware of alternative pain management strategies. The lack of education leads to dependence on unhealthy and poor coping mechanisms for their pain such as reliance on pain medication, cigarettes and sedentary behaviour.

‘I know as a human being if I could, but I won’t get rid of it until the day I go up then I could say I’m rid of the pain. I don’t believe it will end.’ **(>20 years diagnosed)**

‘When I have pain I light a cigarette. I’m still struggling to give that up. It is a problem.’ **(>20 years diagnosed)**

### ‘There’s not much support from the clinic’

Some of the participants expressed their frustration with the services they receive from their PHC clinic. The complaints mostly involved the lack of resources and long waiting times. Participants complained that the clinic often would run out of medicine and scheduled outreaches of medical officers were sporadic. They felt that they were not receiving adequate treatment as they waited long for surgeries and were not referred to appropriate health care providers. This is concerning as most of the areas had a visiting dietician and only two participants were referred for consultation and only one participant expressed to have received a comprehensive rehabilitation programme with multiple visits to the physiotherapist.

‘And then they don’t even have tablets here, or nothing else. Then I must just walk back home… The doctor was supposed to come, but he didn’t come and I still have not heard the result of the X-rays.’ **(4 years diagnosed)**

‘I was probably on the waiting list [for joint replacement surgery] for ten years before they [performed the surgery]… ten years.’ **(>20 years diagnosed)**

Participants also felt unsatisfied with the attention and support they receive from the health care personnel. It appears to lack person-centred care with minimal collaboration with individuals to address their needs.

‘Yes. I feel they should show more interest. I don’t know how to say this, but they are giving me too little.’ **(13 years diagnosed)**

‘If I don’t ask for the results they don’t tell me.’ **(5 years diagnosed)**

‘There’s not much support from the clinic. Because you see, when you get there then they ask you what’s the problem but they don’t do anything about it.’ **(4 years diagnosed)**

In addition, there was a prominent reference to the pharmacological management strategies provided by the health care personnel as being the only treatment they are receiving. This deterred one patient from going back to the clinic.

‘That’s it. Only painkillers for my legs.’**(>20 years diagnosed)**

‘…then they merely told me it’s arthritis and prescribed painkillers. Then I didn’t go again.’**(12 years diagnosed)**

### ‘I don’t feel myself anymore’

Most of the participants have suffered a significant restriction in participation in major life activities due to a reduction in their mobility. Participants were unable to complete meaningful activities which in turn affect their sense of self-worth.

‘I don’t feel myself anymore. Four years ago I could still do everything, but I feel with the arthritis a part of my life is missing because the things I could do, I can’t do anymore … Ooo, I long for it. I did all my work myself. My garden and everything. It’s a little neglected now because I need someone to do that for me.’ **(5 years diagnosed)**

‘I struggle to do it … Because visitors could come and then they’ll see that my house is still not cleaned. I always kept it tidy.’ **(>20 years diagnosed)**

Participants commented on their need to feel good again and to participate in the activities they used to enjoy without this constant concern for their pain. This appeared to stimulate anxiety in their lives, affecting their mental wellbeing. Participants appeared disempowered and expressed the sense of hope that is lost.

‘But I want to do something. I don’t like to just sit and do nothing. So I’m 66 now. I will be 67 and one would like to have a few minutes of feeling good… You worry if you want to work in your garden or want to do anything else at home and you worry whether you will be able to this and that tomorrow again.’ **(>20yrs diagnosed)**

‘I have to sort of drag my legs when I have to do something. Like sweeping I still can’t do… But because I’m alone, I have to do it… and it’s difficult you know… you may be poor, my father used to say, but you don’t need to be untidy. That’s what I always remember. It’s a really a struggle for me.’ **(>20 years diagnosed)**

In summary, the results highlights an interlink between health care system barriers leading to poor patient education and lack of empowerment which inevitably affects the social inclusion and mental wellbeing of the individuals with knee OA. These results are important for contextualisation and improvement of rehabilitation and health care service delivery, especially in a rural context.

## Discussion

The current study aimed to explore the rehabilitation needs as expressed by individuals with knee OA living in rural Western Cape, South Africa, in order to identify the gaps in services offered by the PHC health care system and inform rehabilitation programmes in these settings.

In this study, the PHC was identified as the fundamental barrier to the health-related wellbeing and empowerment of these individuals. Similar to other studies done in South Africa, participants in this study expressed their frustrations with long waiting times, the shortage of medication and the communication issues with the staff at the clinic (Selman *et al.*, 2009; Dookie and Singh, 2012; Visagie and Schneider, 2014). The lack of person-centred care led to a negative attitude towards the PHC system and discouraged them to seek further medical attention. Visagie and Schneider (2014), however, found that the doctors and nurses themselves were frustrated with the system which barely allowed for flexibility in decision-making and were not always user-friendly. In addition, it was also found that that they did not always feel equipped to handle complex situations as they are not experts in all aspects of health care and received little support (Visagie and Schneider, 2014; Lalkhen and Mash, 2015). This is possibly amplified by the lack of time the doctor/nurse has with the patient due to the high patient load in the PHC facilities in South Africa, which in turn reveals the failure of the system and does not necessarily speak to the incompetence of individual health care practitioners (Selman *et al.*, 2009; Bateman, 2012; Cobbing *et al.*, 2014; Major-Helsloot *et al.*, 2014). In addition, Dube *et al*. (2017) found that nursing staff in a South African township lacked the educational material they desired and had no structured plan for self-management education on non-communicable diseases. This leads to conflicting and disorganised information shared with the patients, creating misconceptions and confusion.

An additional barrier identified was the human resource constraints within the PHC system of this particular setting which only allows rehabilitation staff to visit a rural area once a month (Bateman, 2012; Dookie and Singh, 2012; Major-Helsloot *et al.*, 2014). In this study, the bulk of services were delivered by the nursing staff or the doctor, frequent referral for corticosteroid injections and possible surgical management, with occasional referral to rehabilitation professionals. This poor referral pattern has been found in other studies where physicians manage patients themselves and showed a high rate of referral to orthopaedic surgeons for surgery (Brand *et al.*, 2014; Thorstensson *et al.*, 2015). The lack of referral to physiotherapy/dietetics for participants of this study is most likely the main contributing factor for the lack of self-management amongst these individuals. A lack of familiarity with the services or benefits offered by rehabilitation professionals by PHC staff is an additional factor to be considered. It could be argued that education on the role and the benefit of the programmes and approaches offered by rehabilitation professionals could improve referral. Nonetheless, that does not account for patients not being included in the decision-making process or the lack of evidence-based practice used by health care providers, which is still an unfortunate reality of the current health care system and its clinical practice (Major-Helsloot *et al.*, 2014; Lalkhen and Mash, 2015).

Another concern was the prevailing use of pharmacological treatment strategies as the only pain management option at the PHC facility, with a take-home message for the individual that not much could be done for them. Similar findings by Major-Helsloot *et al.* (2014) revealed that 90% of patients only received pain medication from their PHC facility, which would suggest that the PHC system is uninformed about alternative pain management strategies and currently not equipped to provide them, regardless of the condition. This highlights the need for disease-specific information to empower patients in self-management strategies.

The participants in this study expressed that the content of the current disease-related information and advice received were inconsistent and lacked depth. Previous studies emphasised the positive effect of knowledge and insight on the ability of an individual to manage a progressive disorder and to function in one’s daily life (Selman *et al.*, 2009; De Rezende, 2017). Information and knowledge has the capacity to reduce your pain, improve compliance with exercise and most importantly, it could improve your health-seeking behaviour (Brosseau *et al.*, 2010; Mann and Gooberman-Hill, 2011; Cobbing *et al.*, 2014). Similarly to this study, a report from Uganda found that at times the only source of information for a patient was a magazine article or a family member/friend, which could lead to misconceptions and the circulation of myths (Selman *et al.*, 2009). It is also concerning that the knowledge of the health care professionals also appeared questionable as they contributed to the myth that the disease is due to old age and that little can be done for them. This contributed to further misconceptions and did not encourage lifestyle changes or health-seeking behaviour in participants from previous studies (Clarson *et al.*, 2013; Osterås *et al.*, 2013; Spitaels *et al.*, 2017; Ali *et al.*, 2018). This is a major concern which could be addressed with the dissemination of evidence-based guidelines to all PHC staff in a user-friendly manner to update the knowledge of health care professionals and provide quality care.

Social isolation is known amongst people with chronic pain, influencing their participation in social- and community-related activities (Schulman-Green *et al.*, 2016; Makris *et al.*, 2017). Persistent pain and limited functional capacity results in low quality of life and mental wellbeing (Araujo *et al.*, 2016), and the loss of independence and lack of participation in meaningful activities had an emotional and social impact on the lives of the participants in this study. Similar findings in a systematic review by Wallis *et al.*, (2019) showed that people living with knee OA has limited ability to stay socially connected due to reduced mobility which impacted their participation in social and leisure activities and lead to emotional distress such as anxiety and loss of self-worth.

The results of this study place focus on how system barriers amplify the lack of person-centred care, leading to poor quality of life for people living with knee OA. This is particularly true for individuals living in rural areas, who are affected by various social, financial, structural and service delivery constraints, often leading to a cycle of poverty and disability (Luong *et al.*, 2012; Republic of South Africa, Department of Health, 2015; Sherry, 2015). This study advocates that health care system barriers need to be addressed in order to equip the PHC staff with knowledge and resources for delivering person-centred care and in turn improving the health related quality of life of individuals living with knee OA.

Therefore, comprehensiveness and consistency of PHC service delivery in these settings need to be addressed to deliver a multidisciplinary approach to empower these individuals with knowledge to manage their symptoms and improve their participation. This is in accordance with the World Health Organization (WHO) service delivery framework (World Health Organization, 2002). One unique enabler of the PHC setting is the availability of mid-level rehabilitation workers which could be used in concert with rehabilitation services to empower communities. However, this service is not yet available in all communities of South Africa and should be strongly considered for improving the rehabilitation service delivery capacity.

## Conclusion

The rehabilitation needs of individuals with knee OA in rural areas advocate for addressing barriers in rural PHC system, such as adequate human resources, referral systems and continuity of care. This will allow for a comprehensive, person-centred and context-specific multidisciplinary approach focused on empowering individuals with knee OA through disease-specific education, improving functional participation and symptom management strategies. This could improve the social inclusion and mental wellbeing of individuals living with knee OA.

## Limitations

This study has certain limitations which should be considered when interpreting the findings. This study was only conducted in randomly chosen rural areas of the Western Cape. These rural areas are different from other rural communities in South Africa; therefore, the results can only be considered for similar settings such as those visited in this study. The snowball recruitment method resulted in possible sampling bias as well as gender bias, as only one male participant was recruited using this method. It is also acknowledged that the translation of the transcriptions from Afrikaans to English could have altered the meaning of some of the sentences. Certain Afrikaans expressions were lost in translation, as no similar English word or phrase exists. The translation of the transcripts was done for the purpose of reporting the findings in English.

## References

[ref1] Ali SA , Walsh KE and Kloseck M (2018) Patient perspectives on improving osteoarthritis management in urban and rural communities. Journal of Pain Research 11, 417–425. doi: 10.2147/JPR.S150578. 29503578 PMC5826243

[ref3] Araujo ILA , Castro MC , Daltro C and Matos MA (2016) Quality of life and functional independence in patients with osteoarthritis of the knee. Knee Surgery & Related Research 28, 219–224. doi: 10.5792/ksrr.2016.28.3.219. 27595076 PMC5009047

[ref5] Bateman C (2012) ‘One size fits all’ health policies crippling rural rehab – therapists. South African Medical Journal 102, 200–208. doi: http://0-www.samj.org.za. 22616111

[ref6] Brand CA , Harrison C , Tropea J , Hinman RS , Britt H and Bennell K (2014) Management of osteoarthritis in general practice in Australia. Arthritis Care and Research 66, 551–558. doi: 10.1002/acr.22197. 24127305

[ref7] Britten N (2006) Qualitative interviews In Pope, C and Mays, N , editors, Qualitative research in health care, Third edition. London: BMJ books, 12–20.

[ref8] Brosseau L , Wells GA , Pugh AG , Smith CA , Rahman P , Alvarez-Gallardo IC , Toupin-April K , Loew L , De Angelis G , Cavallo S , Taki J , Marcotte R , Fransen M , Hernandez-Molina G , Kenny GP , Regnaux JP , Lefevre-Colau MM , Brooks S , Laferriere L , McLean L and Longchamp G (2010) Ottawa Panel evidence-based clinical practice guidelines for patient education in the management of osteoarthritis. Health Education Journal 71, 397–451. doi: 10.1177/0017896911419346.

[ref9] Clarson LE , Nicholl BI , Bishop A , Edwards JJ , Daniel R , and Mallen CD (2013) Monitoring osteoarthritis: a cross-sectional survey in general practice. Clinical Medicine Insights: Arthritis and Musculoskeletal Disorders 6, 85–91. doi: 10.4137/CMAMD.S12606. 24324351 PMC3855255

[ref10] Cobbing S , Hanass-Hancock J and Deane M (2014) Physiotherapy rehabilitation in the context of HIV and disability in KwaZulu-Natal, South Africa. Disability and Rehabilitation 36, 1–8. doi: 10.3109/09638288.2013.872199. 24383469

[ref11] Conaghan PG , Dickson J and Grant RL (2008) Guidelines: care and management of osteoarthritis in adults: summary of NICE guidance. BMJ 336, 502–503. doi: 10.1136/bmj.39490.608009.AD. 18310005 PMC2258394

[ref12] Creswell JW , Hanson WE , Clark Plano VL and Morales A (2007) Qualitative research designs. The Counseling Psychologist, 35, 236–264. doi: 10.4135/9781849208826.n4i.

[ref13] Dookie S and Singh S (2012) Primary health services at district level in South Africa: a critique of the primary health care approach. BMC Family Practice 13, 2–5. 22748078 10.1186/1471-2296-13-67 PMC3403923

[ref14] Dube L , Rendell-Mkosi K , Van den Broucke S , Bergh AM and Mafutha NG (2017) Self-management support needs of patients with chronic diseases in a South African township: a qualitative study. Journal of Community Health Nursing 34, 21–31. doi: 10.1080/07370016.2017.1260983. 28156143

[ref16] Glyn-Jones S , Palmer AJ , Agricola R , Price AJ , Vincent TL , Weinans H and Carr AJ (2015) Osteoarthritis. The Lancet 386, 376–387. doi: 10.1016/S0140-6736(14)60802-3. 25748615

[ref17] Gómez-Olivé FX , Thorogood M , Clark B , Kahn K and Tollman S (2013) Self-reported health and health care use in an ageing population in the Agincourt sub-district of rural South Africa. Global Health Action 6, 181–192. doi: 10.3402/gha.v6i0.19305. PMC3556700 23364087

[ref18] Hochberg MC , Altman RD , April KT , Benkhalti M , Guyatt G , McGowan J , Toweed T , Welch V , Wells G , Tugwell P and American College of Rheumatology (2012) American College of Rheumatology 2012 recommendations for the use of nonpharmacologic and pharmacologic therapies in osteoarthritis of the hand, hip, and knee. Arthritis Care and Research 64, 465–474. doi: 10.1002/acr.21596. 22563589

[ref19] Hsieh H-F and Shannon SE (2005) Three approaches to qualitative content analysis. Qualitative Health Research SAGE Publications 15, 1277–1289. doi: 10.1177/1049732305276687. 16204405

[ref20] Lalkhen H and Mash R (2015) Multimorbidity in non-communicable diseases in South African primary healthcare. South African Medical Journal 105, 134. doi: 10.7196/SAMJ.8696. 26242533

[ref21] Luong MLN , Cleveland RJ , Nyrop KA and Callahan LF (2012) Social determinants and osteoarthritis outcomes. Aging Health 8, 413–437. doi: 10.2217/ahe.12.43. 23243459 PMC3519433

[ref22] Major-Helsloot ME , Crous LC , Grimmer-Somers K and Louw QA (2014) Management of LBP at primary care level in South Africa: up to standards?. African Health Sciences 14, 698–706. doi: 10.4314/ahs.v14i3.28. 25352891 PMC4209660

[ref23] Makris UE , Higashi RT , Marks EG , Fraenkel L , Gill TM , Friedly JL and Reid MC (2017) Physical, emotional, and social impacts of restricting back pain in older adults: a qualitative study. Pain Medicine (Malden, Mass.) 18, 1225–1235. doi: 10.1093/pm/pnw196. PMC5914385 27516362

[ref24] Mann C and Gooberman-Hill R (2011) Health care provision for osteoarthritis: concordance between what patients would like and what health professionals think they should have. Arthritis Care and Research 63, 963–972. doi: 10.1002/acr.20459. 21387574

[ref25] Mayosi BM , Flisher AJ , Lalloo UG , Sitas F , Tollman SM and Bradshaw D (2009) Health in South Africa 4 The burden of non-communicable diseases in South Africa The Lancet 374, 934–947. doi: 10.1016/S0140-6736(09)61087-4. 19709736

[ref26] Nelson AE , Allen KD , Golightly YM , Goode AP and Jordan JM (2014) A systematic review of recommendations and guidelines for the management of osteoarthritis: the Chronic Osteoarthritis Management Initiative of the U.S. Bone and Joint Initiative. Seminars in Arthritis and Rheumatism 43, 701–712. doi: 10.1016/j.semarthrit.2013.11.012. 24387819

[ref27] O’Brien BC , Harris IB , Beckman TJ , Reed DA and Cook DA (2014) Standards for reporting qualitative research: a synthesis of recommendations. Academic Medicine 89, 1245–1251. doi: 10.1097/ACM.0000000000000388. 24979285

[ref28] Osterås N , Garratt A , Grotle M , Natvig B , Kjeken I , Kvien TK and Hagen KB (2013) Patient-reported quality of care for osteoarthritis: development and testing of the osteoarthritis quality indicator questionnaire. Arthritis Care and Research 65, 1043–1051. doi: 10.1002/acr.21976. 23401461

[ref29] Pope C , Ziebland S and Mays N (2006) Analyising qualitative data. In *Qualitative research in health care*, third edition. BMJ Books, 63–81. 10.1136/bmj.320.7227.114 PMC1117368 10625273

[ref30] Prieto-Alhambra D , Judge A , Javaid MK , Cooper C , Diez-Perez A and Arden NK (2014) Incidence and risk factors for clinically diagnosed knee, hip and hand osteoarthritis: influences of age, gender and osteoarthritis affecting other joints. Annals of the Rheumatic Diseases 73, 1659–1664. doi: 10.1136/annrheumdis-2013-203355. 23744977 PMC3875433

[ref31] Rabiee F (2004) Focus-group interview and data analysis. Proceedings of the Nutrition Society 63, 655–660. doi: 10.1079/PNS2004399. 15831139

[ref32] Republic of South Africa, Department of Treasury (2012) *2011 local government budgets and expenditure review: 2006/07–2012/13* Available at: http://www.treasury.gov.za/publications/igfr/2011/lg/02.%202011%20LGBER%20-%20Final%20-%2013%20Sept%202011%20(renumbered).pdf.

[ref33] Republic of South Africa, Department of Health (2015) Framework and strategy for disability and rehabilitation services in South Africa: 2015–2020. Available at: http://ilifalabantwana.co.za/wp-content/uploads/2016/07/Framework-25-may_1_3.docx.

[ref51] De Rezende MU , De Farias FES , Da Silva CAC , Cernigoy CHA and De Camargo OP (2017) Objective functional results in patients with knee osteoarthritis submitted to a 2-day educational programme: A prospective randomized clinical trial. BMJ Open Sport and Exercise Medicine 2, e000200. doi: 10.1136/bmjsem-2016-000200. PMC5569271 28879035

[ref34] Schulman-Green D , Jaser SS , Park C and Whittemore R (2016) A metasynthesis of factors affecting self-management of chronic illness. Journal of Advanced Nursing 72, 1469–1489. doi: 10.1111/jan.12902. 26781649 PMC4891247

[ref35] Selman L , Higginson IJ , Agupio G , Dinat N , Downing J , Gwyther L , Mashao T , Mmoledi K , Moll AP , Sebuyira LM , Panajatovic B and Harding R (2009) Meeting information needs of patients with incurable progressive disease and their families in South Africa and Uganda: multicentre qualitative study. BMJ (Clinical Research ed.) 338, b1326. doi: 10.1136/bmj.b1326. PMC3273778 19386672

[ref36] Shenton AK (2004) Strategies for ensuring trustworthiness in qualitative research projects. Education for Information 22 (February), 63–75. doi: 10.1111/j.1744-618X.2000.tb00391.x.

[ref37] Sherry K (2015) Disability and rehabilitation: essential considerations for equitable, accessible and poverty-reducing health care in South Africa. South African Health Review 2014/2015 89–100.

[ref100] Spencer LJ , Degu A , Kalkidan HA , Solomon MA , Christiana A , Nooshin A , Hedayat A , Foad A , Jemal A , Ahmed A et al. (2018) Global, regional, and national incidence, prevalence, and years lived with disability for 354 diseases and injuries for 195 countries and territories, 1990–2017: a systematic analysis for the Global Burden of Disease Study 2017. The Lancet 392, 1789–1858. 10.1016/S0140-6736(18)32279-7 PMC6227754 30496104

[ref38] Spitaels D , Vankrunkelsven P , Desfosses J , Luyten F , Vershueren S , Van Assche D , Aertgeerts B and Hermens R (2017) Barriers for guideline adherence in knee osteoarthritis care: a qualitative study from the patients’ perspective. Journal of Evaluation in Clinical Practice 23, 165–172. doi: 10.1111/jep.12660. 27859970

[ref39] Thorstensson CA , Garellick G , Rystedt H and Dahlberg LE (2015) Better management of patients with osteoarthritis: development and nationwide implementation of an evidence-based supported osteoarthritis self-management programme. Musculoskeletal Care 13, 67–75. doi: 10.1002/msc.1085. 25345913

[ref40] Tong A , Sainsbury P and Craig J (2007) Consolidated criteria for reporting qualitative research: a 32-item checklist for interviews and focus groups. International Journal for Quality in Health Care 19, 349–357. 17872937 10.1093/intqhc/mzm042

[ref41] Visagie S and Schneider M (2014) Implementation of the principles of primary health care in a rural area of South Africa. African Journal of Primary Health Care & Family Medicine 6, E1–E10. doi: 10.4102/phcfm.v6i1.562. PMC4502891 26245391

[ref43] Wallis JA , Taylor NF , Bunzli S and Shields N (2019) Experience of living with knee osteoarthritis: a systematic review of qualitative studies. BMJ Open 9, 1–11. doi: 10.1136/bmjopen-2019-030060. PMC6773287 31551381

[ref50] Western Cape Government (2014) *Information on the Rural Areas Act,* Western Cape Government, viewed 18 May 2017. Available at: https://www.westerncape.gov.za/general-publication/information-rural-areas-act.

[ref44] World Health Organization (WHO) (2002) The World Health Organization Report 2002: reducing risks, promoting healthy life. WHO Library Cataloguing-in Publication Data, p. 232. Available at: https://apps.who.int/iris/bitstream/handle/10665/42510/WHR_2002.pdf?sequence=1.

[ref45] World Health Organization (WHO) (2003) International Classification of Funcitoning, Disability and Health. World Health Organization (Version 2.1a, Clinician Form), pp. 1–15. Available at: http://www.who.int/classifications/icf/icfchecklist.pdf?ua=1.

